# Differential Regional and Subtype-Specific Vulnerability of Enteric Neurons to Mitochondrial Dysfunction

**DOI:** 10.1371/journal.pone.0027727

**Published:** 2011-11-16

**Authors:** Andreu Viader, Elizabeth C. Wright-Jin, Bhupinder P. S. Vohra, Robert O. Heuckeroth, Jeffrey Milbrandt

**Affiliations:** 1 Department of Genetics, Washington University School of Medicine, St. Louis, Missouri, United States of America; 2 Department of Pediatrics and Department of Developmental, Regenerative and Stem Cell Biology, Washington University School of Medicine, St. Louis, Missouri, United States of America; 3 Department of Biology, University of Central Arkansas, Conway, Arkansas, United States of America; University of South Florida, United States of America

## Abstract

Mitochondrial dysfunction is a central mediator of disease progression in diverse neurodegenerative diseases that often present with prominent gastrointestinal abnormalities. Gastrointestinal dysfunction in these disorders is related, at least in part, to defects in the enteric nervous system (ENS). The role of mitochondrial deficits in ENS neurodegeneration and their relative contribution to gastrointestinal dysfunction, however, are unclear. To better understand how mitochondrial abnormalities in the ENS influence enteric neurodegeneration and affect intestinal function, we generated mice (Tfam-ENSKOs) with impaired mitochondrial metabolism in enteric neurons and glia through the targeted deletion of the mitochondrial transcription factor A gene (Tfam). Tfam-ENSKO mice were initially viable but, at an early age, they developed severe gastrointestinal motility problems characterized by intestinal pseudo-obstruction resulting in premature death. This gastrointestinal dysfunction was caused by extensive, progressive neurodegeneration of the ENS involving both neurons and glia. Interestingly, mitochondrial defects differentially affected specific subpopulations of enteric neurons and regions of the gastrointestinal tract. Mitochondrial deficiency-related neuronal and glial loss was most prominent in the proximal small intestine, but the first affected neurons, nitrergic inhibitory neurons, had the greatest losses in the distal small intestine. This regional and subtype-specific variability in susceptibility to mitochondrial defects resulted in an imbalance of inhibitory and excitatory neurons that likely accounts for the observed phenotype in Tfam-ENSKO mice. Mitochondrial dysfunction, therefore, is likely to be an important driving force of neurodegeneration in the ENS and contribute to gastrointestinal symptoms in people with neurodegenerative disorders.

## Introduction

Gastrointestinal dysfunction is a prevalent symptom in neurologic and systemic diseases associated with neurodegeneration. Constipation, for example, is the most widely recognized non-motor symptom in people with Parkinson's disease (PD), the second most common neurodegenerative disorder in industrialized countries [1], [2]. Decreased frequency of bowel movements is in fact one of the earliest signs of PD, predating the development of the classic motor symptoms, sometimes by many years [3]–[5]. In addition, impaired gastric emptying is estimated to affect the majority of patients with PD and complicates treatment by interfering with levodopa absorption, which can only be absorbed once it reaches the small intestine (SI) [1], [6], [7]. Similarly, up to 75% people with diabetes mellitus, a systemic metabolic disease associated with progressive neuronal damage [8], experience a variety of gastrointestinal symptoms ranging from diarrhea to severe gastroparesis and constipation [9]. Gastrointestinal dysfunction in these neurodegenerative disorders is related, at least in part, to abnormalities and cell loss in the enteric nervous system (ENS) [9]–[11], the complex network of neurons and glia that innervates the gut and controls intestinal function. A better understanding of the pathophysiology of neurodegeneration within the ENS could therefore be relevant to the treatment of patients with diseases characterized by neuron loss with prominent gastrointestinal symptoms.

Mitochondria are now thought to be critical mediators of disease progression in diverse disorders such as PD and diabetes. Studies primarily focusing on the central nervous system (CNS) have established that mitochondrial dysfunction is involved in both the initiation and propagation of disease processes that eventually result in neuron death [12], [13]. A growing body of evidence indicates that mitochondrial defects may similarly contribute to neurodegeneration in the ENS. Consistent with this notion, rodent models of PD induced by mitochondrial toxins are characterized by ENS pathology and cell loss, even at doses below those necessary to cause CNS pathology [14]–[17]. Indeed, the ENS appears to be particularly susceptible to mitochondrial dysfunction compared to other tissues. This is best exemplified by the fact that primary inherited mitochondrial disorders, a heterogeneous group of complex multisystem diseases, commonly include gastrointestinal symptoms. As is the case in PD, symptoms of gastrointestinal dysfunction can precede other presentations of mitochondrial deficits and, in some cases (e.g. mitochondrial neurogastrointestinal encephalomyopathy, or MNGIE) may be the most prominent manifestation of the disease [18]. The role of mitochondrial abnormalities in ENS neurodegeneration and its relative contribution to gastrointestinal dysfunction, however, remain poorly understood.

With the goal of elucidating how mitochondrial abnormalities in the ENS contribute to enteric neurodegeneration and affect gastrointestinal function, we generated mice with impaired mitochondrial metabolism in neurons and glia of the ENS. These Tfam-ENSKO mice were generated by tissue-specific deletion of the gene encoding mitochondrial transcription factor A (*Tfam*), which is required for mitochondrial DNA (mtDNA) transcription and replication [19]. We show that normal mitochondrial function in the ENS is essential for the survival of both enteric neurons and glia as well as for maintenance of normal gastrointestinal motility. Interestingly, we found that mitochondrial dysfunction differentially affected specific subpopulations of enteric neurons and, most surprisingly, specific regions of the gastrointestinal tract. Mitochondrial deficiency-related neuronal and glial loss was most prominent in the proximal SI, but nitrergic, inhibitory neurons were less severely affected in this region and, instead, were most affected in the distal SI. This regional and subtype variability of enteric neurons appears to directly correlate to the phenotype observed in Tfam-ENSKO mice, with dilation in the proximal SI and relative constriction in the distal SI. Mitochondrial dysfunction in the ENS and the regional- and subtype-specific vulnerabilities of enteric neurons are likely contributors to the gastrointestinal symptoms of patients suffering from some neurodegenerative disorders.

## Results

### *Cre*-mediated deletion of Tfam results in mitochondrial abnormalities in the ENS

To study how mitochondrial dysfunction in the ENS affects gastrointestinal function and contributes to enteric neurodegeneration, we generated mice with disrupted mitochondrial metabolism in both enteric neurons and glia (Tfam-ENSKOs). For this purpose we used a previously developed mouse with *loxP*-flanked *Tfam* alleles (*Tfam* ^loxP^) [19]. Tfam is a mitochondrial protein encoded by nuclear DNA that is essential for mtDNA maintenance, copy number regulation and transcription [19], [20]. Previous studies have shown that *cre*-mediated deletion of *Tfam* in *Tfam^loxP^* homozygous mice results in severe tissue-specific mtDNA depletion and mitochondrial respiratory chain deficiency [19], [21]–[25]. The tissue-specific deletion of *Tfam* is, therefore, an effective way to induce mitochondrial dysfunction in a selected population of cells.

**Figure 1:**
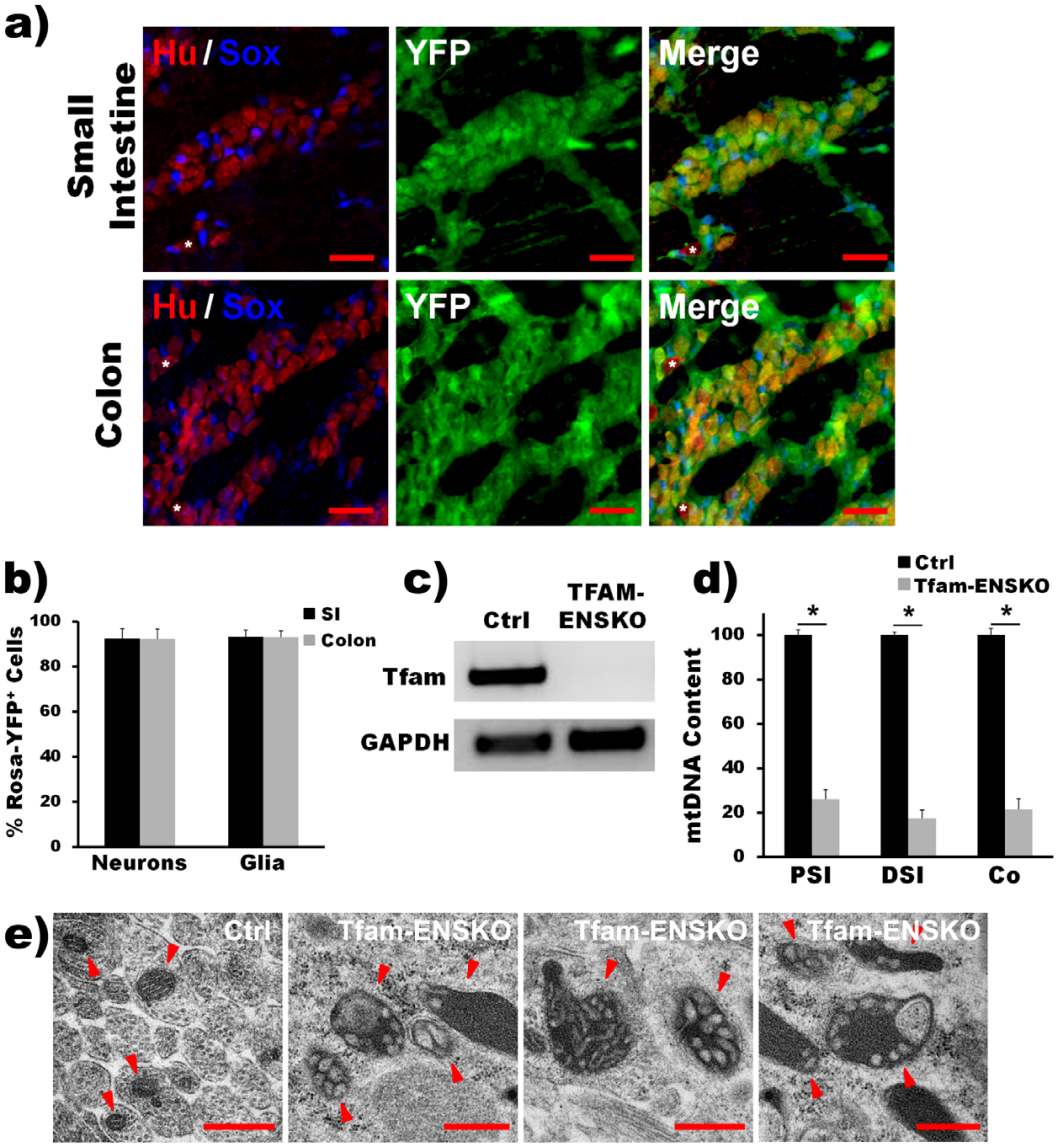
CNP-Cre excises Tfam in enteric neurons and glia and disrupts ENS mitochondria in Tfam-ENSKO mice. ***a*** *)* YFP fluorescence in 2-day-old Rosa26-YFP/CNP-Cre mice overlaps (merge) with enteric neurons (HuC/HuD^+^ cells, Hu, red) and glia (Sox-10^+^ cells, Sox, blue). Excision-dependent YFP fluorescence is visible in the majority of enteric neurons and glia in all gut regions. Asterisks indicate the occasional non-recombined, non-YFP^+^ cell. Scale bars: 30 µm. ***b*** *)* Quantification of the percent of YFP^+^ neurons (% of HuC/HuD^+^ and YFP^+^ cells/total HuC/HuD^+^ cells) and glia (% of Sox-10^+^ and YFP^+^ cells/total Sox-10^+^ cells) in different gut regions confirms high efficiency of CNP-Cre mediated recombination in the ENS. n = 3 mice per genotype. ***c*** *)* RT-PCR of Tfam transcript demonstrates the complete excision of *Tfam* in YFP^+^ FACS selected myenteric neurons and glia in YFP/Tfam-ENSKO mice at 7 weeks of age. n = 3 mice per genotype. ***d*** *)* qRT-PCR results show depletion of mtDNA content in YFP^+^ FACS-selected myenteric neurons and glia in 7 week old YFP/Tfam-ENSKO mice. mtDNA content was significantly reduced in YFP/Tfam-ENSKO mice in all regions examined at this age (*, p<0.01). Reported values are normalized to nuclear DNA content and ratio of mtDNA to nuclear DNA in control animals was set at 100; n = 3 mice per genotype. ***e*** *)* Electron micrographs of myenteric plexus mitochondria (arrowheads) in 7 week old control and Tfam-ENSKO mice. Tfam-ENSKO myenteric neurons and glia contain abundant abnormal, enlarged mitochondria with dilated and distorted cristae. Scale bars, 500 nm.

We mated *Tfam* ^loxP^ mice to mice expressing *cre*-recombinase under the control of the C*np* promoter (CNP-Cre) [26]. *Cnp* encodes the enzyme 2′, 3′-cyclic nucleotide 3′-phosphodiesterase (CNP), a commonly used marker for myelin-forming glia. However, we inadvertently discovered that the *Cnp* promoter also drives the expression of *cre*-recombinase in the ENS and thereby induces recombination in the majority of enteric neurons and glia. Consistent with this fortuitous observation, CNP has been reported to be highly expressed in gut neural crest stem cells [27]. Indeed, when we crossed CNP-Cre mice with Cre-inducible Rosa26-YFP reporter animals, YFP fluorescence was visible in over 90% of enteric neurons and glia throughout the gut (Fig. 1a and b).

Crossing of Tfam-ENSKO mice to Cre-inducible Rosa26-YFP reporter animals (YFP/Tfam-ENSKOs) showed that *Tfam* was efficiently excised in all enteric neurons and glia in which we observed *Cnp*-mediated expression of *cre-*recombinase (as visualized by YFP fluorescence). When we isolated YFP-positive enteric neurons and glia from 7 week old YFP/Tfam-ENSKO mice, we could not detect the *Tfam* allele by RT-PCR analysis (Fig. 1c). Given the extent and high efficiency of the *Cnp* promoter-driven expression of *cre*-recombinase, we conclude that the mating of *Tfam* ^loxP^ to CNP-Cre mice resulted in animals that lacked Tfam in the majority of enteric neurons and glia.

To determine the functional effect of deleting *Tfam* in the ENS, we next assessed mtDNA copy number in YFP-positive enteric neurons and glia from 7 week old YFP/Tfam-ENSKO and YFP/control mice. Tfam has an essential role in the maintenance and replication of mtDNA [19], [20] and previous reports have described severe mtDNA depletion following tissue-specific excision of Tfam from a cell of interest [19], [21]–[25]. Consistent with this, we found an 80% reduction in total mtDNA content in enteric neurons and glia isolated from different regions throughout the gut of YFP/Tfam-ENSKO mice (Fig. 1d).

In previous studies using *Tfam* ^loxP^ mice, the depletion of mtDNA following *Tfam* excision has been shown to induce severe respiratory chain deficiency, since the mitochondrial genome encodes 13 subunits that are essential components of the electron transport chain. In addition, Tfam deficiency-induced mitochondrial dysfunction is accompanied by abnormalities in mitochondrial morphology [19], [21]–[25], [28]. Therefore, to further confirm the enteric neuron- and glia-specific disruption of mitochondria in Tfam-ENSKO mice we examined the enteric nervous system by electron microscopy. Abundant abnormal and enlarged mitochondria with distorted cristae were found specifically within enteric neurons and glia of Tfam-ENSKOs but not of control littermates (Fig. 1e). Together, our results confirm that by deleting *Tfam* in enteric neurons and glia we were able to generate mice with disrupted mitochondria in the ENS.

### Tfam-ENSKOs develop progressive gastrointestinal dysfunction characterized by intestinal pseudo-obstruction

**Figure 2:**
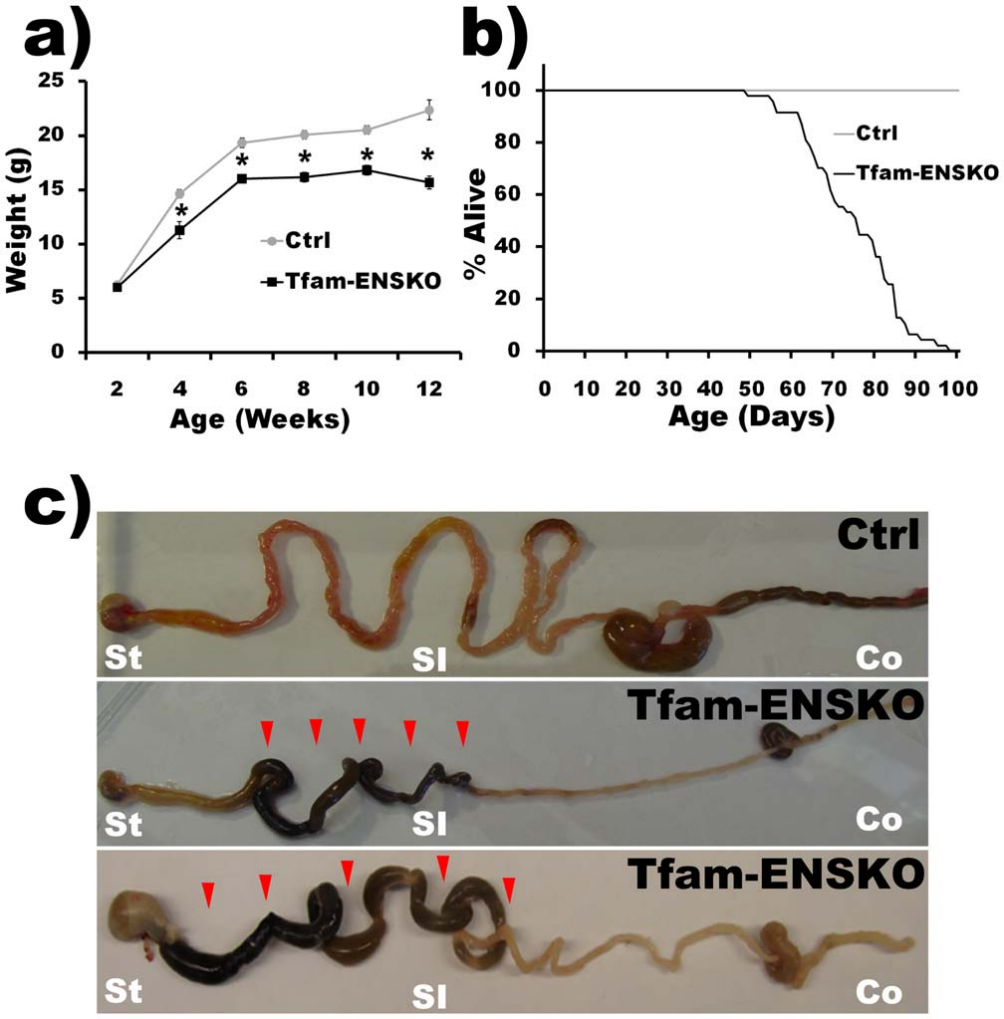
Tfam-ENSKOs develop progressive GI dysfunction characterized by intestinal pseudo-obstruction. ***a*** *)* Growth curve of Tfam-ENSKO mice and control littermates. Tfam-ENSKOs are initially indistinguishable from control mice, but begin to exhibit poor growth at 4 weeks of age (n≥10 mice per genotype at each time point). ***b*** *)* Survival curve of Tfam-ENSKO mice and control littermates. Tfam-ENSKO mice die prematurely beginning at 60 days old. By 90 days, nearly all Tfam-ENSKO mice are dead. n = 47 mice. ***c*** *)* Photograph of gastrointestinal tract of 11-12 week old control and Tfam-ENSKO mice depicts their typical appearance. Arrowheads indicate regions of dilation and accumulation of luminal contents. St: stomach, SI: small intestine, Co: colon.

Tfam-ENSKO mice were viable and born at the expected Mendelian ratios and, for the first 2 weeks of life, they were indistinguishable from their control littermates. After 2 weeks of age, however, Tfam-ENSKO mice displayed signs of poor growth and, by 4 weeks of age, they were significantly smaller than their control littermates (Fig. 2a). In addition, Tfam-ENSKO mice developed abdominal distention at about 6-8 weeks of age, which, together with poor growth, suggests gastrointestinal dysfunction. After 8 weeks of age, the health of Tfam-ENSKO mice deteriorated rapidly and the majority of the animals died by 12 weeks of age (Fig. 2b).

Dissection of late stage (i.e. 10–12 weeks old) Tfam-ENSKO mice consistently revealed massive dilation within the proximal small bowel along with relative contraction of the distal small bowel (Fig. 2c). The region of transition from dilated proximal small bowel to the narrower distal small bowel was consistently located within the mid-small bowel, with variable accumulation of luminal contents in the proximal small bowel and stomach. No stenosis or mechanical cause of the obstruction could be found and the mice did not have malrotation or other anatomic explanations for the obstruction. Proximal to the transition zone, in the region of dilation, the bowel was filled with dark-colored luminal contents and the bowel wall appeared to be stretched thinner than in controls (Fig. 2c). Luminal contents were absent distally and no stool pellets could be found in the colon or rectum. The distal small intestine and colon in Tfam-ENSKOs generally appeared to have a smaller diameter than in control mice. Therefore, we conclude that disrupted mitochondrial metabolism in enteric neurons and glia in Tfam-ENSKO mice results in significant gastrointestinal dysfunction and dysmotility.

### Tfam-ENSKO mice develop progressive neuronal degeneration with distinct regional vulnerabilities

**Figure 3:**
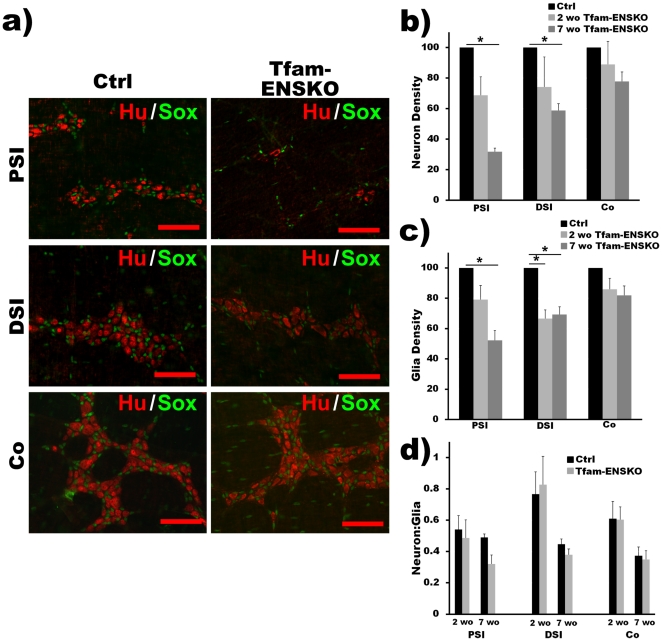
Tfam-ENSKO mice display progressive degeneration of neurons and glia with distinct regional vulnerabilities. ***a*** *)* Representative images of HuC/HuD (neurons, red) and Sox-10 (glia, green) immunohistochemistry in three regions of myenteric plexus of 7 week old control and Tfam-ENSKO mice. Loss of both HuC/HuD^+^ neurons and Sox-10^+^ glia is apparent in Tfam-ENSKOs at this age. Scale bars, 100 µm. ***b*** *)* Quantification of total myenteric neuron density in Tfam-ENSKOs expressed relative to control littermates at 2 and 7 weeks of age. For 2 weeks old, n = 6 (Ctrl) and n = 4 (Tfam-ENSKO). For 7 weeks old, n = 3 for each genotype. ***c*** *)* Quantification of glial density in Tfam-ENSKOs expressed relative to control littermates at 2 and 7 weeks of age. For 2 weeks old, n = 6 (Ctrl) and n = 4 (Tfam-ENSKO). For 7 weeks old, n = 3 for each genotype. ***d*** *)* Neuron-to-glia ratio in ENS of Tfam-ENSKOs and control littermates at 2 and 7 weeks of age demonstrates that relative cell loss is equivalent for both neurons and glia. For 2 weeks old, n = 6 (Ctrl) and n = 4 (Tfam-ENSKO). For 7 weeks old, n = 3 for each genotype. PSI: proximal small intestine, DSI: distal small intestine, Co: colon.

To test the hypothesis that ENS defects underlie the bowel abnormalities and early death observed in Tfam-ENSKO mice, we used whole mount immunohistochemical methods to evaluate ENS structure. We initially examined total myenteric neuron density using immunohistochemistry for HuC/HuD, a widely used pan-neuronal marker that labels the cell body of all neurons within the myenteric plexus [29]. Two week old Tfam-ENSKO mice had a lower mean neuron density in all regions examined compared to control animals, but these differences were not statistically significant (p>0.1 in all cases, Fig. 3b and Table 1). This suggests that the ENS develops normally in these animals and is consistent with the healthy appearance of Tfam-ENSKOs at this age.

**Table 1:** Myenteric Plexus Cell Density.

		Total neuron density (Cells/mm^2^)				Glial cell density (Cells/mm^2^)				Neuron-to-Glia Ratio (Neurons/Glia)				NADPH-d+ Neuron Density (Cells/mm^2^)				% of total neurons that are NADPH-d^+^			
Region	Age (weeks)	Ctrl	Tfam-ENSKO	% of Ctrl	*p* value	Ctrl	Tfam-ENSKO	% of Ctrl	*p* value	Ctrl	Tfam-ENSKO	% of Ctrl	*p* value	Ctrl	Tfam-ENSKO	% of Ctrl	*p* value	Ctrl	Tfam-ENSKO	% of Ctrl	*p* value
PSI	2	1221±156	839±148		0.133	2316±126	1831±216		0.071	0.54±0.09	0.49±0.12		0.706	303±24	188±26	62%	0.030	25	22		N/A
DSI	2	2355±381	1747±461		0.339	3142±239	2094±176	67%	0.012	0.77±0.14	0.83±0.18		0.794	574±28	350±46	61%	0.014	24	20		N/A
Co	2	2113±286	1879±317		0.607	3633±372	3117±268		0.343	0.61±0.11	0.60±0.08		0.961	578±73	557±93		0.860	27	30		N/A
PSI	7	235±23	74±5	31%	0.003	479±31	250±31	52%	0.007	0.49±0.02	0.32±0.06		0.054	82±6	55±4	67%	0.021	36±5	75±12	208%	0.038
D SI	7	329±18	194±14	59%	0.004	744±55	515±38	69%	0.027	0.45±0.03	0.38±0.04		0.400	91±9	36±5	40%	0.007	28±2	19±2	68%	0.035
Co	7	548±85	426±33		0.258	1409±48	1153±87		0.062	0.37±0.06	0.35±0.06		0.782	147±8	110±7	75%	0.027	21±4	26±1		0.288

Myenteric plexus neuron and glial cell density and ratios in 2 and 7-week-old Tfam-ENSKO and control mice. n = 3 for each genotype for all analyses of 7 week old mice. For 2 week old mice, n = 6 (Ctrl) or 4 (Tfam-ENSKO) for total neuron density, glial density, and neuron to glia ratio and n = 3 for NADPH-d^+^ neuron density. N/A, not applicable; due to small size of tissue samples at 2 weeks of age, staining for both HuC/HuD^+^ (total) and NADPH-d^+^ (nitrergic) could not be performed on a single animal and therefore statistical analysis of the proportion of total neurons that are NADPH-d^+^ was not possible. PSI: proximal small intestine, DSI: distal small intestine, Co: colon.

We next examined the myenteric plexus of 7 week old Tfam-ENSKO and control mice. At this age, Tfam-ENSKO mice begin to show subtle phenotypic abnormalities (i.e. poor growth) but appear healthy, do not display signs of gastrointestinal obstruction, and lack intestinal dilation (Fig. 2a and b). Despite their healthy appearance, at 7 weeks of age Tfam-ENSKO mice had a 68% decrease in myenteric neuron density in the proximal SI, and a 41% decrease in the distal SI (Fig. 3a and b and Table 1). Surprisingly, even though the extent of *Tfam* excision appeared equal in all regions analyzed at 7 weeks of age (Fig. 1), we found no difference in colon total neuron density between Tfam-ENSKOs and control littermates at this age (Fig. 3a and b and Table 1). In fact, enteric neuron density in the colon of Tfam-ENSKOs remained normal even at late pathological stages (e.g. 10-12 weeks old), when enteric neurodegeneration in more proximal regions was profound (data not shown). Thus, mitochondrial dysfunction in the ENS results in progressive neurodegeneration with marked differences in regional vulnerability to neuronal loss.

### Glial degeneration parallels that of neurons in Tfam-ENSKOs

A growing body of literature now implicates glial dysfunction in many neurodegenerative diseases traditionally thought to be neuron autonomous. In animal models of amyotrophic lateral sclerosis, PD, and Huntington's disease among others, glia-specific abnormalities alter disease onset and progression (for a review see [30]). To address the role of enteric glia in the mitochondria-related gastrointestinal dysfunction of Tfam-ENSKO mice, we assessed the effect of Tfam-depletion on enteric glia using Sox-10 immunohistochemistry. We first examined glial density in the myenteric plexus of 2 week old mice and observed very little difference between Tfam-ENSKOs and littermate controls except in the distal SI (Fig. 3c and Table 1). By 7 weeks of age, however, Tfam-ENSKO mice had significantly reduced glial cell density in both the proximal and distal SI (Fig. 3a and 3c and Table 1). However, as is the case for HuC/HuD^+^ neurons, the glial cell density in the colon of Tfam-ENSKO mice remained normal at 7 weeks of age (Fig. 3a and 3c and Table 1).

The changes in glial cell density in Tfam-ENSKO mice, therefore, seemed to parallel that of neurons, involving the same regions and a similar proportion of cells. We examined this in more detail by comparing the ratio of neurons to glia in control and Tfam-ENSKO mice. As expected from the absence of either neuron or glial cell loss in 2 week old Tfam-ENSKOs, we found no difference in the neuron to glia ratio in any of the regions examined compared to control animals (Fig. 3d and Table 1). Interestingly, despite extensive neuron and glia loss in 7 week old Tfam-ENSKOs, the ratio of neurons to glia in all regions examined remained comparable to that of control littermates (Fig. 3d and Table 1). These results suggest that enteric neurons and glia are equally susceptible to mitochondrial defects in Tfam-ENSKO mice.

### Early and differential loss of nitrergic inhibitory neurons in Tfam-ENSKO mice

**Figure 4:**
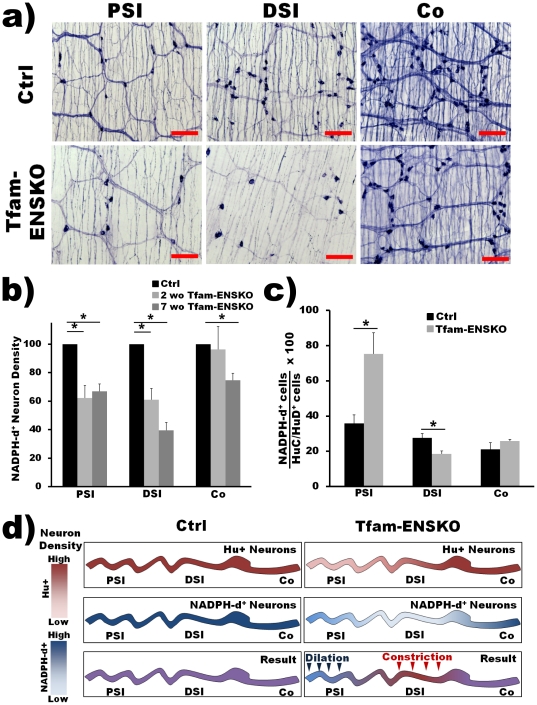
Early and differential loss of nitrergic inhibitory neurons in Tfam-ENSKO mice. ***a*** *)* Representative images of NADPH-d stained myenteric plexus show inhibitory neurons in the proximal SI and distal SI in 7 week old Tfam-ENSKO mice. Scale bar, 150 µm. ***b*** *)* Quantitative analysis of NADPH-d^+^ neuron density in Tfam-ENSKOs relative to control littermates at 2 and 7 weeks of age. n = 3 for each genotype at each age. ***c*** *)* Ratio of nitrergic neurons to total neurons in 7 week old Tfam-ENSKO and control mice. n = 3 for each genotype. PSI: proximal small intestine, DSI: distal small intestine, Co: colon. ***d*** *)* Diagram depicting how the imbalance of inhibitory neurons to excitatory neurons in each region in Tfam-ENSKOs could produce the observed proximal SI dilation and distal SI constriction. In Tfam-ENSKO mice, greater total neuron loss (Tfam-ENSKO top panel) relative to NADPH-d^+^ neuron loss (Tfam- ENSKO middle panel) would result in increased inhibitory input and dilation of the proximal SI (Tfam-ENSKO bottom panel). Greater NADPH-d^+^ neuron loss (Tfam-ENSKO middle panel) relative to total neuron loss (Tfam-ENSKO top panel) would result in decreased inhibitory input and constriction (and pseudoobstruction) of the distal SI of Tfam-ENSKO mice (bottom panel). Color intensity represents neuronal density with WT density set as the most intense color in all regions of the bowel.

Intestinal motility and peristalsis depend on the balance between ENS excitatory and inhibitory inputs which together produce the rhythmic coordination of contraction and relaxation necessary to propel luminal contents [31]. The apparent constriction observed in the distal bowel of Tfam-ENSKO mice, as well as the dilation proximal to this point (Fig. 2c), suggested the possibility of an imbalance between these inputs. To address how enteric neuron loss in Tfam-ENSKO mice affects the balance between inhibitory and excitatory inputs, we examined the number and proportion of nitric oxide (NO)-producing inhibitory neurons in the myenteric plexus by NADPH diaphorase (NADPH-d) staining [32]. The proportion of NADPH-d^+^ neurons in different gut regions is normally within a narrow range that maintains the balance between ENS excitatory and inhibitory inputs and allows for normal intestinal motility. As early as 2 weeks of age, the NADPH-d^+^ neuron density in the proximal and distal SI in Tfam-ENSKO mice was significantly lower than in controls (Fig. 4b and Table 1). These data suggest early preferential loss of nitrergic inhibitory neurons relative to other neuronal subtypes.

As the pathologic changes in the ENS of Tfam-ENSKO mice progressed, the loss of NADPH-d^+^ neurons became more pronounced and, moreover, by 7 weeks of age the percentage of NADPH-d^+^ neurons varied quite dramatically between different regions of the gut. NADPH-d^+^ neuron loss was greatest in the distal SI with a 60% decrease, but significant decreases were also found in the proximal SI and colon (33% and 25%, respectively; Fig. 4a and b and Table 1). Interestingly, the observed loss of nitrergic neurons did not parallel the loss of total neurons in each region (Fig. 3a and b), causing significant differences in the relative proportion of NADPH-d^+^ neurons to total neurons (i.e. changes in inhibitory input) throughout the bowel (Fig. 4c and Table 1). In 7 week old Tfam-ENSKOs, NADPH-d^+^ neurons accounted for 75% of total neurons in the proximal SI compared to 36% in controls (Fig. 4c and Table 1). In contrast, in the distal SI, the proportion of NADPH-d^+^ inhibitory neurons was lower than in control animals, (19% in Tfam-ENSKOs, 28% in controls; Fig. 4c and Table 1). In the colon, there was no significant difference in the ratio of nitrergic to total neurons between control and Tfam-ENSKO mice (Fig. 4c and Table 1). This concurrent increase in relative abundance of inhibitory inputs in the proximal SI along with the decrease in the ratio of NADPH-d^+^ to total neurons in the distal SI of Tfam-ENSKOs may thus account for the proximal dilation and distal constriction observed in the late pathology of these mice (Fig. 4d).

### Axonal degeneration is a key feature of enteric neuron loss in Tfam-ENSKOs and may precede cell body loss

**Figure 5:**
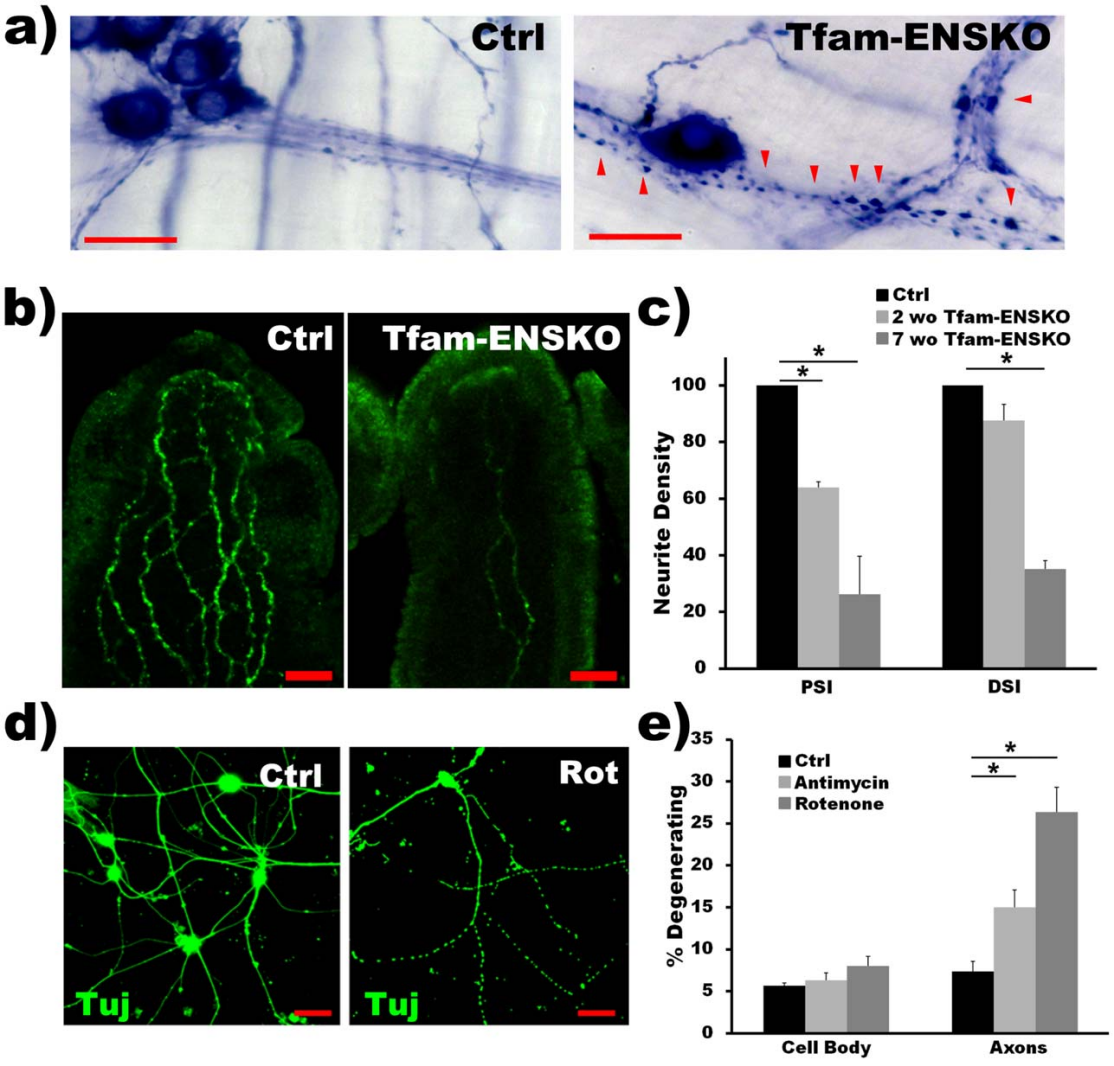
Axonal degeneration is present in Tfam-ENSKO mice and may precede enteric neuron loss. ***a*** *)* Tfam-ENSKO mice show noticeable neurite blebbing (arrowheads) in the proximal and distal SI at 7 weeks of age. Scale bars, 25 µm. ***b, c*** *)* Representative images (b) and quantification (c) of TuJ1^+^ neurites extending into SI villi of 7 week old control and Tfam-ENSKO mice. n = 3 for each genotype. Scale bars, 40 µm.***d, e*** *)* Representative images (d) and quantification (e) of axonal fragmentation (TuJ1, green) and cell body loss (ethidium homodimer, staining not shown) in cultured enteric neurons after treatment with the mitochondrial inhibitors rotenone or antimycin. Images correspond to untreated or rotenone-treated enteric neurons. n =  duplicate wells for each condition from 3 independent assays. Scale bars, 100 µm.

During our examination of NADPH-d^+^ neurons, we observed extensive and prominent blebbing in neuronal projections in the myenteric plexus of Tfam-ENSKO mice (Fig. 5a). In the CNS and PNS, axon blebbing has been recognized as a sign of axonal degeneration, a central component of many neurodegenerative diseases, which can precede and sometimes cause neuronal death [33]. To assess the role of axon degeneration in the neuron loss observed in Tfam-ENSKOs we first examined the neuronal projections, or neurites, extending into the villi in the proximal and distal SI. As early as two weeks of age, Tfam-ENSKO mice displayed signs of neurite loss within intestinal villi, although at this age neurite degeneration was limited to the proximal SI (Fig. 5c and Table 2). Neurite loss worsened over time, and by 7 weeks of age the number of neurites in the villi of Tfam-ENSKO mice was significantly reduced in both the proximal and distal SI (Fig. 5b and c and Table 2) suggesting that loss of neuronal projections could contribute to subsequent neuron loss.

**Table 2:** Villus Neurite Density.

		Neurites/villus			
Region	Age (weeks)	Ctrl	Tfam-ENSKO	% of Ctrl	*p* value
PSI	2	4.25±0.18	2.72±0.09	64%	0.002
DSI	2	3.63±0.25	3.18±0.2		0.235
PSI	7	10±1.3	2.6±0.23	26%	0.006
DSI	7	12±1.6	4.1±0.35	34%	0.011

Density of neuronal projections in intestinal villi in 2 and 7 week old Tfam-ENSKO and control mice. n = 3 for each genotype and age. PSI: proximal small intestine, DSI: distal small intestine, Co: colon.

Because we are unable to easily identify the cell of origin for neurites in the villi, we also quantified neuronal fiber density for NADPH-d^+^ fibers in the myenteric plexus of 7 week old mice. We found no difference in the density of large fiber bundles (i.e., primary fibers; [34]) in any region examined between Tfam-ENSKOs and controls (Table 3). In contrast, small fiber density (i.e., secondary and tertiary fibers; [34]) was significantly lower (44%) in the proximal SI of Tfam-ENSKOs mice, but was similar to controls in the distal SI and colon (Table 3). Note, however, that what we count as “small fibers” or “large fiber bundles” are actually one or more tightly fasciculated neurites. In Tfam-ENSKO mice both “small fibers” and “large fiber bundles” appear significantly thinner than in control animals at 7 weeks of age (Fig. 4a and 5a), suggesting that our counting method for NADPH-d+ neuronal projections significantly underestimates neurite loss in the mutant mice. Because of these limitations, we devised an alternate method to evaluate the effect of mitochondrial dysfunction on enteric neuron and neurite degeneration *in vitro*.

**Table 3:** Nitrergic Fiber Density.

		NADPH-d^+^ Bundles/mm^2^				NADPH-d^+^ Fibers/mm^2^				NADPH-d^+^ Fibers/neuron			
Region	Age (weeks)	Ctrl	Tfam-ENSKO	% of Ctrl	*p* value	Ctrl	Tfam-ENSKO	% of Ctrl	*p* value	Ctrl	Tfam-ENSKO	% of Ctrl	*p* value
PSI	7	19±0.5	14±2		0.073	312±18	174±32	56%	0.020	3.9±0.6	3.1±0.5		0.208
DSI	7	22±3	19±0.5		0.700	298±25	233±6		0.100	3.4±1.4	6.8±4.8		0.098
Co	7	25±1	25±1		0.844	583±56	527±5		0.378	4.0±0.7	4.8±0.2		0.099

Myenteric NADPH-d^+^ fiber density and number of NADPH-d^+^ fibers per neuron in 7 week old Tfam-ENSKO and control mice. n = 3 for each genotype. PSI: proximal small intestine, DSI: distal small intestine, Co: colon.

E12.5 enteric neurons were cultured from control mouse gut and exposed to different inhibitors of the mitochondrial electron transport chain (e.g. rotenone and antimycin). Disrupting mitochondrial respiration induced significant neurite degeneration within 24 hours, yet did not initially affect neuron viability as measured by ethidium homodimer staining (Fig. 5d and e and Table 4). Moreover, even though extended exposure to mitochondrial inhibitors eventually induced enteric neuron death, the proportion of cells showing axonal degeneration remained larger at every time point examined (Table 4). Together, these results suggest that axonal degeneration precedes enteric neuron cell body loss induced by mitochondrial defects.

**Table 4:** *In vitro* Mitochondrial Inhibitor Assay Results.

	Neurons with fragmented axons (%/well)			% of Control		*p* value		Et_2_D^+^ (dead) Neurons (%/well)			% of Control		*p* value	
Time	Veh	Rot	Antim	Rot	Antim	Rot	Antim	Veh	Rot	Antim	Rot	Antim	Rot	Antim
24 hrs	7.3±1.2	26.3±3	15±3.6	360%	205%	0.004	0.03	5.7±0.3	8±1.15	6.3±1.5			0.124	0.5
48 hrs	6.7±1.2	51.7±6.7	12±1	772%	179%	0.003	0.01	6.7±0.3	31±3.6	12±2.6	463%	179%	0.002	0.01
72 hrs	7.7±1.2	83.7±4.7	32±6.8	1087%	416%	<0.001	0.003	7±0.6	58.7±5.2	19.3±3.2	839%	276%	<0.001	0.003

*In vitro* mitochondrial inhibitor assay results from cultured E12.5 immunoselected enteric neurons. n =  duplicate wells for each condition from 3 independent assays. Veh = Vehicle; Rot = Rotenone; Antim = Antimycin

## Discussion

The current understanding of how mitochondrial deficits affect enteric neurons and contribute to ENS neurodegeneration and gastrointestinal dysfunction has been hindered by a lack of animal models. Here we describe a new mouse model with disrupted mitochondrial function in enteric neurons and glia. We show that abnormal mitochondrial metabolism in the ENS causes widespread enteric neurodegeneration and results in severe intestinal pseudo-obstruction, the likely cause of premature death in Tfam-ENSKO mice. Remarkably, mitochondrial dysfunction differentially affected specific subpopulations of enteric neurons and regions of the gastrointestinal tract. This regional and subtype-specific variability resulted in an imbalance of inhibitory and excitatory enteric neurons that likely accounts for the observed phenotype in Tfam-ENSKO mice. Our observations support the hypothesis that damage to the ENS resulting from defects in mitochondrial function may underlie some of the pathophysiology involved in gastrointestinal abnormalities in many human neurodegenerative diseases.

Mitochondria are thought to be critical mediators of neuron loss in diverse neurodegenerative disorders such as PD or diabetes. We show that mitochondrial defects in the ENS cause both neuron and glia cell loss as well as severe intestinal dysmotility. Strikingly, not all regions of the gut were equally affected. The proximal SI suffered the most extensive neuron and glial loss whereas the colon was largely spared (Fig. 3). Furthermore, we observed differential vulnerability among distinct subtypes of enteric neurons. Nitrergic neurons, which are predominantly inhibitory in nature, were lost earliest in the progression of the disease in Tfam-ENSKOs (Fig. 4). These findings cannot be explained by variations in the efficiency of *Tfam* excision or in the resulting effect on mitochondria. *Cnp*-mediated expression of *cre-*recombinase (as visualized by YFP fluorescence) and mtDNA depletion were comparable throughout the bowel of Tfam-ENSKO mice (Fig. 1). The observed variability in sensitivity to mitochondrial defects suggests, therefore, that there are regional and cell type specific differences in metabolic needs for individual enteric neuron populations.

In the CNS, regional- and subtype-specific differences in the vulnerability of neurons to mitochondrial dysfunction are well established; such differences are thought to underlie the preferential loss of striatal neurons in PD or Huntington's disease [35]. Early preferential loss of nitrergic neurons has also been reported in a streptozotocin-induced rat model of diabetic autonomic neuropathy [36]. Similar differences in vulnerability to metabolic insults among enteric neurons had been suggested by an earlier study using a rotenone-induced rat model of PD [16]. The enteric nervous system abnormalities observed in Tfam-ENSKO mice suggest that regional- and subtype-specific differences in the susceptibility of neurons to mitochondrial defects are also present in the ENS and may explain the gastrointestinal presentations of neurodegenerative diseases.

While the mitochondrial dysfunction in Tfam-ENSKO mice is caused by the loss of Tfam, the way in which these mice recapitulate gastrointestinal pathological features often seen in neurodegenerative disorders indicates that there may be broader implications of this work for understanding the bowel dysfunction that accompanies both rare and common human disease. For example, Tfam-ENSKO mice consistently developed intestinal pseudo-obstruction with dilated proximal small bowel and contracted distal bowel reminiscent of that seen in the human mitochondrial disease MNGIE. Our results indicate that this pseudo-obstruction could occur because of an imbalance in the ratio of inhibitory to excitatory inputs in the ENS. In Tfam-ENSKOs such an imbalance arises from a relative preservation of nitrergic inhibitory neurons in the proximal SI and preferential loss of these same neurons in the distal SI of these mice. This is of particular interest, because the severe intestinal pseudo-obstruction resulting in cachexia and death in MNGIE patients has traditionally been attributed to smooth muscle abnormalities, even though mtDNA depletion is also found within enteric neurons in the SI of MNGIE patients [37]. Our results demonstrating that mitochondrial dysfunction in enteric neurons and glia alone is capable of producing very similar pathology, however, suggest that the ENS may play a more prominent role in this mitochondrial disease than previously thought.

While MNGIE is a rare disease, mitochondrial dysfunction is thought to be a common underlying mechanism in both normal human aging [38] as well as in many common diseases such as type 2 diabetes [39]. Defects in gastrointestinal motility frequently cause serious problems in the elderly and in patients with diabetes [9], [40]. These motility defects, similar to what we observed in Tfam-ENSKO mice, have been attributed to enteric neurodegeneration [9], [40]. Moreover, enteric neurodegeneration in models of aging, PD and diabetes have been associated with imbalances in inhibitory and excitatory neurons [40]. As such, understanding the ENS abnormalities in Tfam-ENSKO mice could provide valuable insight into the pathophysiology responsible for gastrointestinal motility disorders involved in numerous disease processes and affecting a large number of people.

In summary, Tfam-ENSKO mice are the first genetic model of enteric nervous system-specific mitochondrial dysfunction and interestingly recapitulate a number of pathological features often seen in human neurodegenerative diseases with prominent gastrointestinal presentations, such as MNGIE, PD or diabetes mellitus. Enteric mitochondrial dysfunction in these diseases is therefore likely to contribute to their gastrointestinal pathology. Moreover, Tfam-ENSKOs revealed a remarkable region- and subtype-specific differential vulnerability of enteric neurons to defects in mitochondrial metabolism. Appropriate management of gastrointestinal dysfunction in patients with neurodegenerative diseases might thus be facilitated by devising therapeutic strategies that improve ENS mitochondrial function and address the differential vulnerability of specific enteric neuron populations.

## Materials and Methods

### Ethics

All studies were reviewed and approved by the Washington University Animal Studies Committee, protocol approval # 20110071 (J.M.) and # 20090190 (ROH).

### Matings of transgenic animals

*Tfam* ^loxP/loxP^ mice [19] and CNP-Cre mice [26] in pure C57Bl/6 backgrounds were crossed to generate Tfam-ENSKO mice (*CNP-Cre* ^+/-^, *Tfam* ^loxP/loxP^) and their control littermates (*CNP-Cre* ^-/-^, *Tfam* ^loxP/loxP^ or *CNP-Cre* ^-/-^, *Tfam* ^+/loxP^). For experiments involving YFP fluorescence-based FACS sorting or imaging of enteric neurons and glia, Tfam-ENSKO mice were crossed to Rosa26-YFP reporter mice [41] to generate YFP/Tfam-ENSKO mice (*Rosa26-YFP* ^+/-^, *CNP-Cre* ^+/-^, *Tfam* ^loxP/loxP^). Mice with transgenic *Rosa26-YFP* and *CNP-Cre* alleles, but with wild type Tfam alleles (YFP/control: *Rosa26-YFP* ^+/-^, *CNP-Cre* ^+/-^, *Tfam* ^+/+^), were used as controls in these experiments. Tfam^loxP/loxP^, CNP-Cre and Rosa26-YFP genotyping were carried out as previously described [19], [26], [41].

### Quantitative ENS analysis

Mice were euthanized by carbon dioxide asphyxiation followed by cervical dislocation. The gastrointestinal tract was removed and enteric whole-mount samples were prepared. Intestines were flushed with ice cold PBS, then opened by cutting along the mesenteric border, flattened and pinned mucosal side down, and fixed using 4% paraformaldehyde for 30 minutes. After fixation, samples were washed with ice cold PBS before isolating the myenteric plexus by peeling off the muscle layers of the intestine. A 6 cm-long intestinal sample was isolated from the proximal SI (measured from the pylorus), distal SI (measured from the ileocecal junction), and distal colon (measured from the anus). In the 2-week-old mice, 3–4 cm long samples were obtained from the same regions as described above. Samples were then cut into 1 to 2 cm segments before storing in 50% glycerol/PBS at −20°C until staining and analysis.

In the 7-week-old mice, sequential 1 cm-long samples of myenteric plexus were stained with NADPH-d [42], biotinylated-HuC/HuD (1∶250; Invitrogen A21272) and Sox-10 (1∶250; Santa Cruz). In the 2-week-old mice, sequential 1-1.5 cm long samples of myenteric plexus were stained with NADPH-d or biotinylated-HuC/HuD and Sox-10, again using the order described above. For the biotinylated-HuC/HuD and Sox-10 double labeling, samples were first labeled by HuC/HuD immunohistochemistry (1∶250; Invitrogen A21272) and then labeled by Sox-10 immunohistochemistry (1∶250; Santa Cruz).

Quantification of neuron or glial cell density was done by counting all cells present within the borders of a 0.5×0.5 mm grid (20x objective lens). For instances in which the cellular density was very high, counts were done using a 40x objective lens (within the borders of a 0.25×0.25 mm grid). An attempt was made to stretch all segments evenly and equally for all samples. All analyses used three to six animals, and counting was done without knowledge of the mouse genotype. Twenty (20) randomly selected fields were counted for each region and data are presented as neurons/mm^2^. Averages for each animal were used to calculate the mean, standard deviation and standard error for each genotype. Neuron-to-glia ratio was determined by dividing the number of neurons by the number of glia present within a single field.

### Villus neurite quantification

Villus preparations were made by cutting along single rows of intestinal villi from full-thickness, pinned samples of proximal and distal small intestine in 70% ethanol. These were then labeled by TuJ1 (1∶10,000; Covance) immunohistochemistry. Villus neurite quantification was determined by counting all TuJ1^+^ projections that crossed a perpendicular line drawn through the middle of each villus. Twenty (20) randomly selected villi were counted for each region and averaged for each animal. At least 3 mice were used to calculate the mean, standard deviation and standard error for each genotype.

### FACS isolation of enteric neurons and glia

Rosa26-YFP^+^ cells were sorted by flow cytometry after isolating and digesting unfixed myenteric plexus from 7-week-old YFP/control and YFP/Tfam-ENSKO mice. A modified version of the protocol published by Schafer et al. [43] was used to isolate adult mouse ENS neurons and glia. Briefly, muscle layers of the bowel containing the myenteric plexus were isolated by dissection as described above, but were not fixed. Samples from the proximal SI, distal SI and colon were cut into 1 mm^2^ pieces in ice-cold PBS, then treated with collagenase (1 mg/ml in DMEM; 37°C, 45 min). After collagenase treatment, tissues were centrifuged at 4,500 rpm for 2 min and collagenase solution was replaced with 0.25% Trypsin in DMEM. Samples were rotated (37°C, 20–30 min) and triturated by pipetting before centrifugation (4,500 rpm for 2 min) to remove supernatant. Cells were diluted in 300 µL FACS buffer (0.002% BSA, 0.001% Sodium Azide, 1 mM EDTA in PBS) and filtered through 70 micron MACS separation filters. Cells were then sorted for YFP expression on a MoFlo Cell Sorter (Beckman Coulter Corp., Fullerton, CA), using 15 p.s.i. and a 120 µm sorting nozzle. YFP fluorescence was captured with a 525/40 optical filter in the cytometer's FL 1 channel. Samples were taken from three mice of each genotype (YFP/Ctrl: *Rosa26-YFP* ^+/-^, *CNP-Cre* ^+/-^, *Tfam* ^+/+^; YFP/Tfam-ENSKO: *Rosa26-YFP* ^+/-^, *CNP-Cre* ^+/-^, *Tfam* ^loxP/loxP^) and a non-YFP^+^ littermate (*Rosa26-YFP* ^-/-^, *CNP-Cre* ^+/-^, *Tfam* ^+/+^ or *Rosa26-YFP* ^-/-^, *CNP-Cre* ^+/-^, *Tfam* ^loxP/loxP^) was used for standardization.

### RNA and DNA preparation and quantitative real-time PCR (qRT-PCR)

RNA and DNA were isolated from FACS sorted enteric neurons and glia from YFP/Tfam-ENSKO mice and YFP/Ctrl littermates. For RNA isolation, cells were lysed in Trizol reagent (Invitrogen) and total RNA prepared according to the manufacturer's protocol. For DNA isolation, cells were digested and DNA isolated using DNeasy Blood and Tissue Kit (Qiagen) according to the manufacturer's protocol. DNA and RNA concentration were quantified using an ND-1000 spectrophotometer (Nanodrop Technologies).

For RT-PCR, cDNA was reverse transcribed from total RNA using M-MLV reverse transcriptase (Invitrogen) and Tfam or GAPDH mRNA were amplified using the following primers (5′-3′): Tfam: F, CAGGAGGCAAAGGATGATTC; R, ATGTCTCCGGATCGTTTCAC; GAPDH: F, TGCCCCCATGTTTGTGATG; R, TGTGGTCATGAGCCCTTCC. mtDNA content was quantified by qRT-PCR using a SYBR green-based detection system on a 7700 Sequence Detector instrument (Applied Biosystems) as described previously [44]. Instead of cDNA, however, 15 ng of total DNA were used per reaction. Primers that recognize a region unique to the mitochondrial genome were used to determine mtDNA content normalized to nuclear DNA content, as determined by a set of primers directed to the genomic locus of Smrt1. The primers used were as follows: mtDNA: F, AAGTCGTAACAAGGTAAGCA; R, ATATTTGTGTAGGGCTAGGG. Nuc.DNA: F, GGGTATATTTTTGATACCTTCAATGAGTTA; R, TCTGAAACAGTAGGTAGAGACCAAAGC

### Cell culture

Enteric neural crest cells were immunoselected from embryonic day 12.5 (E12.5) CD1 mice small bowel and colon using p75^NTR^ antibody (Millipore). Bowel was dissociated with collagenase (1 mg/ml) and dispase (1 mg/ml) to yield a single cell suspension. After p75^NTR^ antibody incubation (Millipore, 1∶1000, 1 h, 4°C) in B27 (Invitrogen) supplemented Neurobasal medium, cells were incubated with goat anti-rabbit coupled paramagnetic beads (1∶50, 1 h, 4°C; Miltenyi Biotec, Bergisch Gladbach, Germany) before separating neural crest-derived cells from unselected cells with a positive selection column (MACS separation columns; Miltenyi Biotec). Immunoselected crest-derived cells were plated at a density of 500 cells per well on poly-d-lysine/laminin-coated 24-well plates in B27 supplemented Neurobasal medium plus 50 ng/ml glial cell line-derived neurotrophic factor (GDNF). In all experiments, inhibitors were added to the medium 24 h after plating using 2.5 µm antimycin (Complex III inhibitor) or 2.5 µm rotenone (Complex I inhibitor). Control wells were treated with DMSO vehicle. All experiments were performed in triplicate. All analyses were performed blinded.

### Neuronal cell body death assay and Immunohistochemistry

Neurons were treated with inhibitors or vehicle control and monitored for cell body damage by ethidium homodimer exclusion (Biotium, Hayward, CA). Ethidium homodimer was added to the cultures at a final concentration of 100 nM and after 30 minutes of incubation cells were washed with PBS and fixed 4% paraformaldehyde. After fixation, cells were washed with PBS and blocked with 5% normal donkey serum in TBST (Tris-buffered saline plus 0.1% Triton X-100) (1 h, 37°C). Cells were incubated in rabbit polyclonal Tuj1 antibody (1∶1000 at 4°C overnight) and antibody binding was visualized with Alexa Fluor 488 conjugated anti-rabbit secondary antibodies (Jackson Immunoresearch Laboratory, 1∶500, 25°C, 1 h).

### Electron Microscopy

Samples for transmission electron microscopy were obtained from 7 week old mice by intracardiac perfusion of Modified Karnovsky's Fixative (2.5% glutaraldehyde, 2% paraformaldehyde in 0.1 mmol/liter cacodylate buffer), followed by isolation of whole mount samples as described above, omitting peeling. Gut samples were pinned in modified Karnovsky's Fixative and post-fixed in 2% OsO_4_ (in 0.1 mmol/L cacodylate buffer). Samples were then dehydrated and embedded in PolyBed 812 (Polysciences Inc., Warrington, PA). 100 nm sections were cut with a Diatome diamond knife and stained with uranyl acetate in 50% methanol and Venable's lead citrate. Transmission EM was performed on a JEOL JEM 1200-EX microscope with AMT Advantage HR (Advanced Microscopy Techniques Corp., Danvers MA) high-speed, wide-angle 1.3 megapixel TEM digital camera.

### Light Microscopy and quantification

Samples were counted using a 10×10 grid counting eyepiece on an Olympus Optical Bx60 microscope or Zeiss Axioskop and an Axiocam digital camera and AxioVision imaging software (Zeiss, Germany). Photoshop 7.0 was used to uniformly adjust contrast and brightness so that digital images appear as they did when observed directly through the microscope.

### Statistical analysis

All values are expressed as mean ± SEM. P values were determined by paired Student's t-test or Mann-Whitney rank sum test using Sigma Plot 11.0 (Systat Software, San Jose, CA). In all applicable figures, an asterisk (*) indicates statistical significance (p value < 0.05).
